# Endothelial glycocalyx during early reperfusion in patients undergoing cardiac surgery

**DOI:** 10.1371/journal.pone.0251747

**Published:** 2021-05-17

**Authors:** Arie Passov, Alexey Schramko, Ulla-Stina Salminen, Juha Aittomäki, Sture Andersson, Eero Pesonen

**Affiliations:** 1 Division of Anaesthesiology, Department of Anaesthesiology Intensive Care and Pain Medicine, University of Helsinki and Helsinki University Hospital, Helsinki, Finland; 2 Department of Cardiac Surgery, Heart and Lung Center, University of Helsinki and Helsinki University Hospital, Helsinki, Finland; 3 Children’s Hospital, Pediatric Research Center, University of Helsinki and Helsinki University Hospital, Helsinki, Finland; Indiana University School of Medicine, UNITED STATES

## Abstract

**Background:**

Experimental cardiac ischemia-reperfusion injury causes degradation of the glycocalyx and coronary washout of its components syndecan-1 and heparan sulfate. Systemic elevation of syndecan-1 and heparan sulfate is well described in cardiac surgery. Still, the events during immediate reperfusion after aortic declamping are unknown both in the systemic and in the coronary circulation.

**Methods:**

In thirty patients undergoing aortic valve replacement, arterial concentrations of syndecan-1 and heparan sulfate were measured immediately before and at one, five and ten minutes after aortic declamping (reperfusion). Parallel blood samples were drawn from the coronary sinus to calculate trans-coronary gradients (coronary sinus–artery).

**Results:**

Compared with immediately before aortic declamping, arterial syndecan-1 increased by 18% [253.8 (151.6–372.0) ng/ml vs. 299.1 (172.0–713.7) ng/ml, p < 0.001] but arterial heparan sulfate decreased by 14% [148.1 (135.7–161.7) ng/ml vs. 128.0 (119.0–138.2) ng/ml, p < 0.001] at one minute after aortic declamping. There was no coronary washout of syndecan-1 or heparan sulfate during reperfusion. On the contrary, trans-coronary sequestration of syndecan-1 occurred at five [-12.96 ng/ml (-36.38–5.15), p = 0.007] and at ten minutes [-12.37 ng/ml (-31.80–6.62), p = 0.049] after reperfusion.

**Conclusions:**

Aortic declamping resulted in extracardiac syndecan-1 release and extracardiac heparan sulfate sequestration. Syndecan-1 was sequestered in the coronary circulation during early reperfusion. Glycocalyx has been shown to degrade during cardiac surgery. Besides degradation, glycocalyx has propensity for regeneration. The present results of syndecan-1 and heparan sulfate sequestration may reflect endogenous restoration of the damaged glycocalyx in open heart surgery.

## Introduction

A mesh of proteoglycans and glycosaminoglycans called the glycocalyx covers the luminal side of the blood vessels [1]. Together with associated plasma proteins, the glycocalyx forms the endothelial surface layer that participates in mechanotransduction and endothelial permeability as well as regulates adhesion of leukocytes and platelets onto the luminal surface [2–4].

Experimental cardiac ischemia-reperfusion (IR) injury causes degradation of the glycocalyx and shedding of its components (syndecan-1 and heparan sulfate) into the blood stream [5, 6]. Many studies indicate that cardiac surgery and cardiopulmonary bypass (CPB) cause degradation of the glycocalyx and consequently increase plasma concentrations of syndecan-1 and heparan sulfate [6–12]. Despite this, to the best of our knowledge, none of these studies provide information on the changes of the glycocalyx related to immediate reperfusion. First, the assessment of the glycocalyx biomarkers after aortic declamping has been done at the end or after CPB and not during early reperfusion. Second, the measurements are made on samples obtained from the systemic circulation. Therefore, the events occurring within the timeframe of immediate reperfusion both in the systemic circulation and in the coronary circulation remain undescribed.

The aim of this study was to investigate the effect of aortic declamping and cardiac reperfusion on the glycocalyx in cardiac surgery. Therefore, we measured concentrations of syndecan-1 and heparan sulfate in patients undergoing aortic valve replacement. Measurements were made in samples from the systemic arterial blood immediately before and repeatedly during first ten minutes after aortic declamping. To assess changes in the coronary glycocalyx, we concomitantly measured syndecan-1 and heparan sulfate in coronary sinus blood and calculated trans-coronary gradients of these biomarkers.

## Patients and methods

The Ethics Committee of Helsinki University Hospital approved the study protocol (Dnro 144/13/03/02/2013). We prospectively recruited 30 patients undergoing aortic valve replacement due to aortic valve stenosis. All patients gave written informed consent before enrolment. The exclusion criteria were coronary artery disease, left ventricular ejection fraction < 30%, atrial fibrillation, systemic glucocorticoid medication or need for perioperative glucocorticoid substitution, immunosuppressive therapy, anti-platelet medication other than aspirin, and anticoagulation before surgery [13].

Anesthesia and CPB were conducted according to our institution`s standards and the detailed protocol has been described previously [13]. In brief, anesthesia was induced with etomidate, alfentanyl and rocuronium and maintained with sevoflurane and infusions of alfentanil and rocuronium. After induction of anaesthesia trans-oesophageal echocardiography was done and myocardial performance index was measured [14]. The CPB circuit was primed with Ringer’s acetate solution. A roller pump and non-pulsatile flow of 2.4 L/min x body-surface-area were used. Mean arterial pressure was maintained at 40–60 mmHg during CPB and at 60–80 mmHg after CPB. Patients were cooled to 33–34 °C. Packed red blood cells were transfused if hemoglobin was < 70 g/L during CPB and < 80 g/L after CPB. Other blood products were used according to clinician`s discretion.

The coronary sinus was cannulated with a balloon tipped 14Fr Retrograde Cardioplegia Catheter (Edwards Lifesciences, Irvine, CA, USA). Correct placement of the catheter was confirmed with trans-esophageal ultrasound and by comparison of partial pressure of oxygen (pO_2_) and hemoglobin oxygen saturation (HbSO_2_) in simultaneously taken pulmonary arterial and coronary sinus blood samples. Lower pO_2_ and HbSO_2_ in the coronary sinus than in the pulmonary artery (i.e. the mixed venous sample) were assumed to indicate correct placement of the coronary sinus catheter. Cardioplegia was induced by antegrade infusion of cold-blood-cardioplegia solution (4:1 cardioplegia-solution-to-blood ratio) double the volume needed for cessation of all cardiac electrical activity but never less than 1,000 ml. For maintenance, retrograde infusion of 300 ml of blood-cardioplegia solution (8:1 cardioplegia-solution-to-blood ratio) every 20 minutes was used.

Blood samples were drawn at six time points: (T1) before induction of anesthesia; (T2) immediately before ischemia (i.e. immediately before aortic cross-clamping); (T3) immediately before reperfusion (i.e. immediately before aortic declamping); (T4) one minute after aortic declamping; (T5) five minutes after aortic declamping; (T6) ten minutes after aortic declamping. The sample was taken from the peripheral arterial cannula at T1 and from the arterial line of the CPB at T2 –T6. Concomitantly with arterial samples at T2 and T4 –T6, parallel blood samples were drawn from the coronary sinus. The samples were immediately divided into separate vacuum tubes containing sodium citrate and transferred to ice-water bath. Thereafter plasma was separated within 20 min by centrifugation at +4C. Plasma was stored in aliquots at -80C. Commercial ELISA kits were used for measurements of syndecan-1 (Diaclone SAS, Besancone, France), heparan sulfate (Elabscience Biotechnology Co.) and heart-type fatty-acid binding protein (Hycult Biotech, Uden, The Netherlands). Heart-type fatty-acid binding protein (HFABP) is a sensitive and rapid biomarker of myocardial injury [15]. In the present study HFABP served as a positive control of dual-sample strategy in detection of trans-coronary phenomena [13].

Data were analyzed with SPSS 23 (IBM Corporation, Armonk, New York, USA) and Graphpad Prims (Graphpad Software LLC, La Jolla, California, USA) programs. The study was observational by nature. While there was no intervention, power analysis for the size of a treatment group was not applicable. Trans-coronary concentration gradients were calculated by subtracting the value of the arterial sample from the value of the coronary sinus sample. Non-parametric approach was used due to small sample size and non-normal distribution of study variables in Shapiro-Wilk test. Friedman test with a post-hoc Wilcoxon signed rank test was used for testing differences as a function of time. For other paired comparisons, Wilcoxon signed rank test was used. Spearman’s test was used for bivariate correlations. P-values < 0.05 were considered statistically significant. Data are expressed as median and interquartile range (IQR) or depicted as line graphs.

## Results

### Patient characteristics and procedural data

Patient characteristics, baseline cardiac function and procedural data are presented in Table 1.

**Table 1:** Patient and procedure data.

Female	15 (50%)
Age (years)	66 (61–72)
Body mass index (kg/m^2^)	26 (24–32)
Body surface area (m^2^)	1.9 (1.7–2.0)
Preoperative creatinine (μmol/L)	77 (69–89)
Preoperative left ventricular ejection fraction (%)	65 (60–69)
Myocardial performance index* (seconds)	0.31 (0.20–0.47)
Cardiopulmonary bypass support time (minutes)	101 (86–117)
Aortic cross-clamping time (minutes)	71 (59–80)

Data are expressed as number (percentage) or median (interquartile range).

*Myocardial performance index was measured after induction of anaesthesia but before surgical incision.

### Verification of the correct placement of the coronary sinus catheter

The results of the verification have been published previously [13]. Before onset of CPB, lower pO_2_ and lower HbSO_2_ in coronary sinus blood compared with the mixed venous blood confirmed the correct placement of the coronary sinus catheter in all but one patient. In this patient, the unverified coronary sinus sample was deleted. The apparently dislodged catheter was repositioned and after reperfusion all samples proved valid and were included.

**Fig 1:**
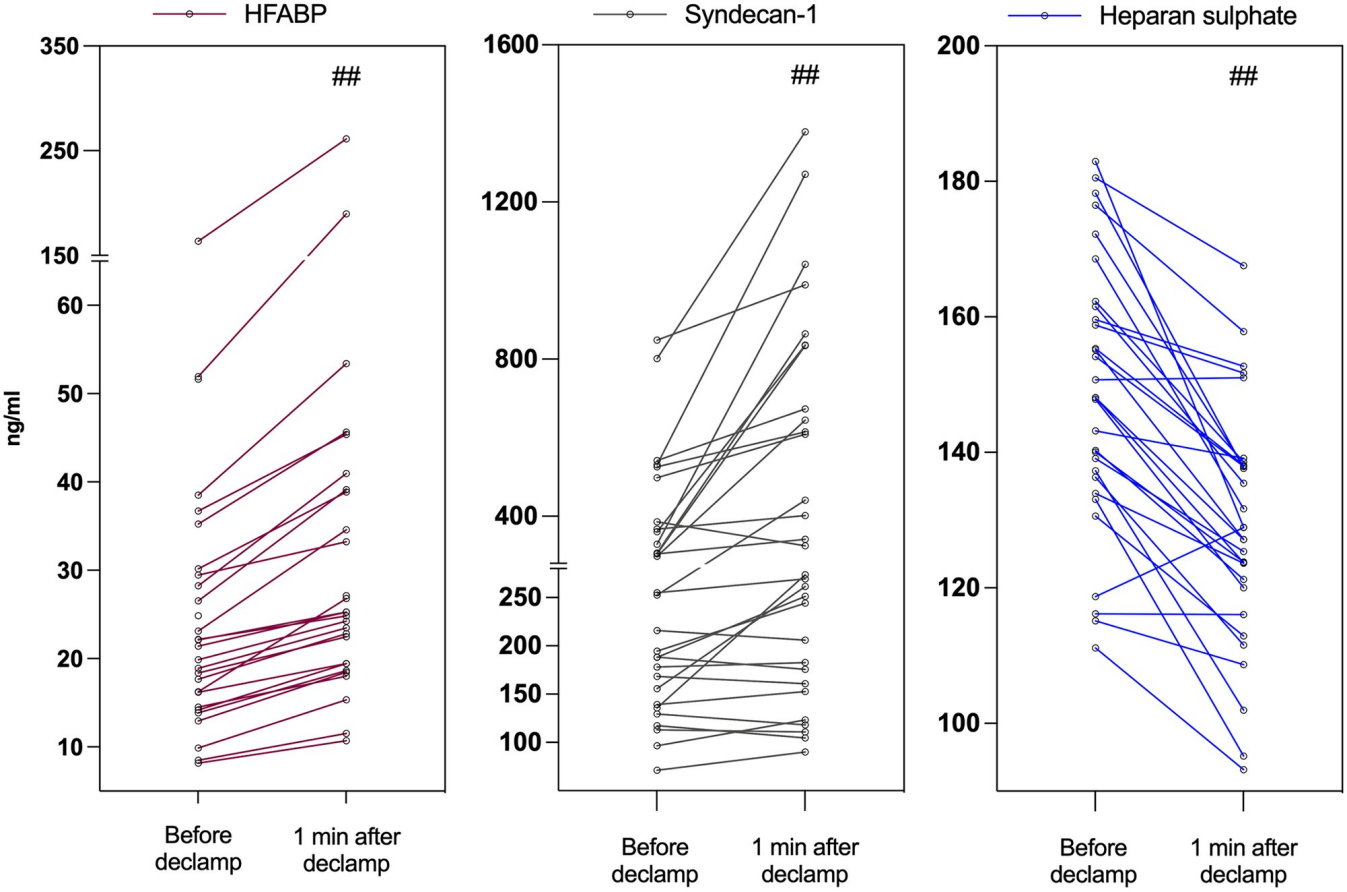
Systemic arterial concentrations of heart-type fatty-acid binding protein, syndecan-1 and heparan sulfate immediately before and at one minute after aortic declamping. Individual values of heart-type fatty-acid binding protein, syndecan-1 and heparan sulfate concentrations in arterial blood immediately before (Before) and at one minute after (1 min after) aortic declapmping. ^#^p < 0.05, ^##^p < 0.001 for Wilcoxon signed rank test (immediately before aortic declamping *vs*. one minute after aortic declamping).

**Fig 2:**
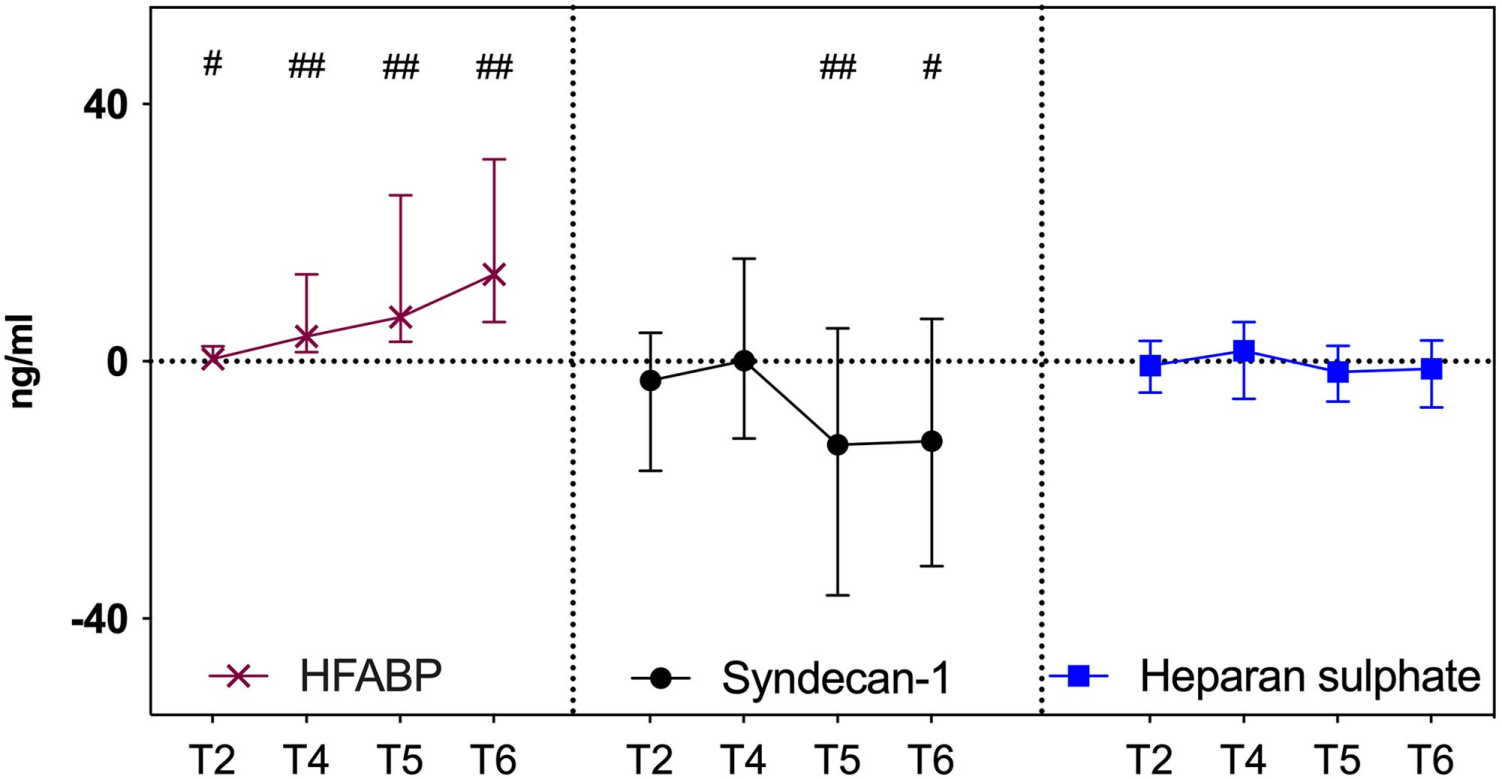
Trans-coronary concentration gradients of heart-type fatty-acid binding protein, syndecan-1 and heparan sulfate during early reperfusion. The median and interquartile range values of trans-coronary concentration gradients of heart-type fatty-acid binding protein, syndecan-1 and heparan sulfate. T2—immediately before aortic cross clamping (i.e. immediately before ischaemia); T4—one minute after aortic declamping; T5—five minutes after aortic declamping; T6—ten minutes after aortic declamping. ^##^p < 0.001 and ^#^p < 0.05 [Wilcoxon signed rank test (coronary effluent blood *vs* arterial blood)].

HFABP served as a positive control for detecting trans-coronary concentration gradients and the results have been published before [13]. Rapid elevation of systemic arterial concentrations of HFABP occurred during reperfusion (T3 vs T4, p = 0.001, Fig 1; T3 vs T5, p < 0.001; T3 vs T6, p < 0,001). The gradients were significant at all time-points (Fig 2).

### Markers of glycocalyx degradation in arterial blood

The median plasma concentration of syndecan-1 in arterial blood changed significantly as a function of time (p < 0.001, Table 2). The increase from the pre-reperfusion value was 18% at one minute after aortic declamping (T3 vs T4, p < 0.001, Fig 1 and Table 2), 69% at five minutes after aortic declamping (T3 vs T5, p < 0.001, Table 2) and 101% at ten minutes after aortic declamping (T3 vs T6, p < 0.001, Table 2). Plasma concentrations of syndecan-1 correlated significantly with CPB time at T5 (R = 0.606, p < 0.001) and T6 (R = 0.562, p = 0.001) but not with ischemia time.

**Table 2:** Concentrations of syndecan-1 and heparan sulfate in systemic arterial blood.

Time-point	Syndecan-1 (ng/ml)	Heparan sulfate (ng/ml)
T1—*before induction of anesthesia*	14.4 (12.1–26.5)	88.5 (85.1–96.8)
T2—*immediately before aortic cross-clamping*	146.1 (82.5–279.7)	82.3 (75.1–86.2)
T3—*immediately before aortic declamping*	253.8 (151.6–372.0)	148.1 (135.7–161.7)
T4—*one minute after aortic declamping*	299.1 (172.0–713.7)^a^	128.0 (119.0–138.2)^a^
T5—*five minutes after aortic declamping*	428.5 (242.2–864.7)^a^	123.4 (110.6–138.4)^a^
T6—*ten minutes after aortic declamping*	509.6 (260.5–820.5)^a^	133.5 (118.8–146.7)^b^

Data are expressed as number median (interquartile range).

^a^ p < 0.001 and

^b^ p = 0.004 for Wilcoxon signed rank test (T3 *vs* T4; T3 *vs* T5 and T3 *vs* T6).

The median concentration of heparan sulfate in systemic blood increased as a function of time (p < 0.001, Table 2). In contrary to the syndecan-1, aortic declamping caused a decrease of 14% in the median concentration of heparan sulfate (T3 vs T4, p < 0.001, Fig 1 and Table 2). After aortic declamping, the median concentration of heparan sulfate remained significantly below the pre-declamping value (T3 vs T5, p < 0.001; T3 vs T6, p = 0.004, Table 2). Heparan sulfate values did not correlate with clinical variables nor with syndecan-1.

### Trans-coronary concentration gradients of markers of glycocalyx degradation

We did not observe any trans-coronary gradients of syndecan-1 either before CPB (T2) or at one minute after aortic declamping (T4) (Fig 2). The latter indicated that there was no cardiac washout of syndecan-1 at one minute after aortic declamping. In contrast, significant negative trans-coronary concentration gradient appeared at five minutes after aortic declamping [-12.96 ng/ml (-36.38–5.15), p = 0.007, Fig 2] indicating syndecan-1 sequestration into the coronary circulation. The gradient remained significant at ten minutes after aortic declamping [-12.37 ng/ml (-31.80–6.62), p = 0.049, Fig 2]. Both these gradients correlated significantly with each other (R = 0.531; p = 0.003). Trans-coronary gradients of syndecan-1 did not correlate with either trans-coronary gradients or systemic values of HFABP. There were no significant gradients of heparan sulfate across the coronary circulation (Fig 2).

## Discussion

The present study is the first to elucidate the impact of ischemia-reperfusion phenomenon on glycocalyx degradation during early reperfusion in clinical open heart surgery. Aortic declamping provoked rapid elevation of systemic levels of extracardiac syndecan-1. While syndecan-1 increased, surprisingly, heparan sulfate decreased. Furthermore, instead of efflux of glycocalyx degradation markers, cardiac reperfusion resulted in trans-coronary sequestration of syndecan-1.

In accordance with previous studies, syndecan-1 concentrations in systemic circulation began increasing already before aortic crossclamping, that is, already before onset of ischaemia [11, 12, 16]. There are at least two potential mechanisms. First, CPB causes complement activation and endotoxin release [17]. Ensuing activation of leukocytes causes release of reactive oxygen species, cytokines and enzymes (matrix metalloproteinases, elastase and heparanase) that degrade the glycocalyx [18–22]. Second, atrial natriuretic peptide (ANP), that degrades the glycocalyx experimentally [23], can contribute to the elevated syndecan-1 levels. In clinical settings, fluid loading [24] and cardiac surgery [16] both cause ANP release and subsequent glycocalyx shedding.

In the present study, we specifically investigated the impact of ischaemia-reperfusion phenomenon and we therefore took samples before aortic declamping and repeatedly during 10 minutes thereafter. We found, that after aortic declamping, syndecan-1 concentrations in the systemic circulation increased by 18% within only one minute. Concomitantly there was no detectable washout of syndecan-1 from the heart. Thus, this elevation must reflect extracardiac washout of previously shed syndecan-1. We speculate the pulmonary vasculature as a probable source of sydnecan-1. First, the pulmonary glycocalyx is thick and sensitive to inflammation [18, 25, 26]. Inflammation is a hallmark of CPB. Inflammatory reaction in the pulmonary vasculature occurs already at one minute after aortic declamping [17, 27]. Second, since 5–20% of the systemic blood flow passes through the lungs after aortic declamping [27], washout of syndecan-1 from pulmonary circulation is probable.

Concurrently with rising systemic syndecan-1 concentrations, the systemic levels of heparan sulfate decreased by 14% during the first minute after aortic declamping and thereafter remained below the pre-reperfusion level. Corroborating our present finding, heparan sulfate decreases in liver transplantation after restoration of the splanchnic and lower body circulation. [28]. Rapid decrease of systemic heparan sulfate due to metabolic clearance within a time interval of only one minute is unlikely. Furthermore, heparan sulfate decreased before protaminization and thus before potential removal of protamine-bound heparan sulfate from the circulation [29]. While there was no retention of heparan sulfate into the heart, sequestration of circulating heparan sulfate must have been extracardiac. We propose that this rapid sequestration of heparan sulfate relates to an endogenous attempt to restore the damaged glycocalyx.

Preclinical and clinical literature supports rapid endogenous restoration of the glycocalyx. First, in experimental studies heparan sulfate and other glycosaminoglycans adhere rapidly onto the damaged glycocalyx [30–32]. Second, in preclinical animal models of hemorrhagic shock, infusion of fresh frozen plasma and albumin restores the glycocalyx within two hours [33–35]. Likewise, in human grade 1 hemorrhage (500 ml blood donation over six minutes), endogenous preservation of the glycocalyx thickness coincided with the decrease in circulating heparan sulfate concentration [36]. Third, intravital microscopy in clinical settings reveals re-thickening of the damaged glycocalyx. In kidney transplantation, re-thickening occurred within 30 minutes of reperfusion [37]. Likewise and importantly, in a study comparing pulsatile and non-pulsatile CPB, glycocalyx was degraded during ischemia in both groups. However, by the time of sternal wound closure, in pulsatile flow group, the thickness of the glycocalyx was almost completely restored [8]. Since *de novo* synthesis of glycocalyx presumably takes several days [38, 39], these data suggest a presence of endogenous mechanism for rapid regeneration of the glycocalyx.

In experimental cardiac I/R model of *ex vivo* perfused Langerdorff heart, local degradation of the glycocalyx causes venous washout of syndecan-1 and heparan sulfate [5, 40]. Similarly, in clinical I/R injury during kidney and liver transplantation, damage to the glycocalyx causes the release of syndecan-1 into the graft vein [28, 37]. Contradicting these previous observations, we did not detect trans-coronary washout of syndecan-1 from the heart. We suspect that intermittent cardioplegia cleansed the coronary circulation and washed syndecan-1 out from the coronary circulation already before onset of reperfusion i.e. before we measured trans-coronary concentration gradients.

While intermittent cardioplegia probably abolished washout of accumulated syndecan-1, at the same time, it revealed trans-coronary sequestration of this proteoglycan during cardiac reperfusion. The fact that coronary syndecan-1 entrapment lasted at least up to ten minutes after the onset of cardiac reperfusion suggests, instead of passive accumulation, an active uptake mechanism. There are at least two potential explanations. First, soluble syndecan-1 can bind to neutrophils [41]. In the very same patient cohort, we have previously observed neutrophil sequestration into the coronary circulation during reperfusion [13]. Therefore, it is possible that the sequestered neutrophils mediate concomitant syndecan-1 sequestration. Alternatively, circulating syndecan-1 may directly attach to the coronary glycocalyx. Indeed, in an *in vitro* model, preassembled complete glycocalyx merged rapidly onto the glycocalyx [42].

During reperfusion, heparan sulfate is released form the graft in kidney transplantation [37] but sequestered in the graft in liver transplantation [28]. In the present study, we did not observe either release or sequestration of heparan sulfate across the coronary circulation. Like with syndecan-1, intermittent cardioplegia probably washed out any heparan sulfate accumulated during ischemia. What comes to the non-existent coronary sequestration of heparan sulfate during reperfusion, there can be only two mutually exclusive explanations. First, it is possible that neither coronary release nor sequestration occurred after intermittent cardioplegia. Second, as glycocalyx seems a dynamic structure, the processes of shedding [5, 37] and sequestration [28] may occur in parallel. In other words, trans-coronary uptake and sequestration of heparan sulfate may be in equilibrium during the timeframe of reperfusion in this study. After all, in a clinical context, any explanations of the present trans-coronary findings of both syndecan-1 and heparan sulfate are forced to remain speculative.

Our study has several strengths. First, exclusion of patients with coronary artery disease abolished confounding by arteriosclerosis on the results. Likewise, pre- and intraoperative glucocorticoids were avoided. Second, we thoroughly confirmed the correct position of the coronary sinus catheter by trans-esophageal ultrasound as well as by paired blood-gas analyses between coronary sinus and mixed venous samples and HFABP analyses between coronary sinus and arterial samples. HFAPB served also as a positive control for the ability to detect trans-coronary concentration differences with our sampling method [13]. There are also limitations. First, we did not directly visualize changes of the glycocalyx with either intravital microscopy or myocardial biopsies. Second, sevoflurane, reducing glycocalyx shedding experimentally [4], possibly influenced the degree of cardiac glycocalyx degradation. As always in cardiac surgery, also heparinization may have affected the results of heparan sulfate. Heparins inhibit heparinase and can potentially affect heparan sulfate shedding [43, 44]. Third, the use of blood products was not standardized and some patients received albumin and fresh frozen plasma during surgery. Finally, experimental studies suggest, that blood pressure levels might affect cardiac syndecan-1 expression [45]. However, here we did not study rapid changes of blood pressure related to aortic declamping.

In conclusion, aortic declamping in open heart surgery is associated with significant extracardiac release of syndecan-1, which is accompanied by momentous extracardiac sequestration of heparan sulphate. Furthermore, syndecan-1 is rapidly sequestered into the coronary circulation during early reperfusion. Thus far, glycocalyx has been thought only to degrade during cardiac surgery. Based on existing literature, however, glycocalyx seems to be a dynamic structure with also a propensity for rapid regeneration [30–32]. Corroborating previous findings, we speculate that, besides degradation, the present results reflect endogenous restoration of the damaged glycocalyx in open heart surgery. The mechanisms and clinical significance of these phenomena remain to be investigated.
